# Antarctic meteorites threatened by climate warming

**DOI:** 10.1038/s41558-024-01954-y

**Published:** 2024-04-08

**Authors:** Veronica Tollenaar, Harry Zekollari, Christoph Kittel, Daniel Farinotti, Stef Lhermitte, Vinciane Debaille, Steven Goderis, Philippe Claeys, Katherine Helen Joy, Frank Pattyn

**Affiliations:** 1 Laboratoire de Glaciologie, Université libre de Bruxelles, Brussels, Belgium; 2 Laboratory of Hydraulics, Hydrology and Glaciology (VAW), ETH Zürich, Zurich, Switzerland; 3 Swiss Federal Institute for Forest, Snow and Landscape Research (WSL), Birmensdorf, Switzerland; 4 Department of Water and Climate, Vrije Universiteit Brussel, Brussels, Belgium; 5 Department of Geography, UR SPHERES, University of Liège, Liège, Belgium; 6 Institut des Géosciences de l’Environnement (IGE), Université Grenoble Alpes/CNRS/IRD/G-INP, Grenoble, France; 7 Department of Earth and Environmental Sciences, KU Leuven, Leuven, Belgium; 8 Department of Geoscience and Remote Sensing, Delft University of Technology, Delft, the Netherlands; 9 Laboratoire G-Time, Université libre de Bruxelles, Brussels, Belgium; 10 Archaeology, Environmental Changes and Geo-Chemistry, Vrije Universiteit Brussel, Brussels, Belgium; 11 Department of Earth and Environmental Sciences, University of Manchester, Manchester, UK

**Keywords:** Climate-change impacts, Cryospheric science

## Abstract

More than 60% of meteorite finds on Earth originate from Antarctica. Using a data-driven analysis that identifies meteorite-rich sites in Antarctica, we show climate warming causes many extraterrestrial rocks to be lost from the surface by melting into the ice sheet. At present, approximately 5,000 meteorites become inaccessible per year (versus ~1,000 finds per year) and, independent of the emissions scenario, ~24% will be lost by 2050, potentially rising to ∼76% by 2100 under a high-emissions scenario.

## Abstract (web summary)

Most of the meteorites on the Earth’s surface are found in Antarctica. Here the authors show that ~5,000 meteorites become inaccessible per year as they melt into the ice due to climate change.

## Main

**Fig. 1:**
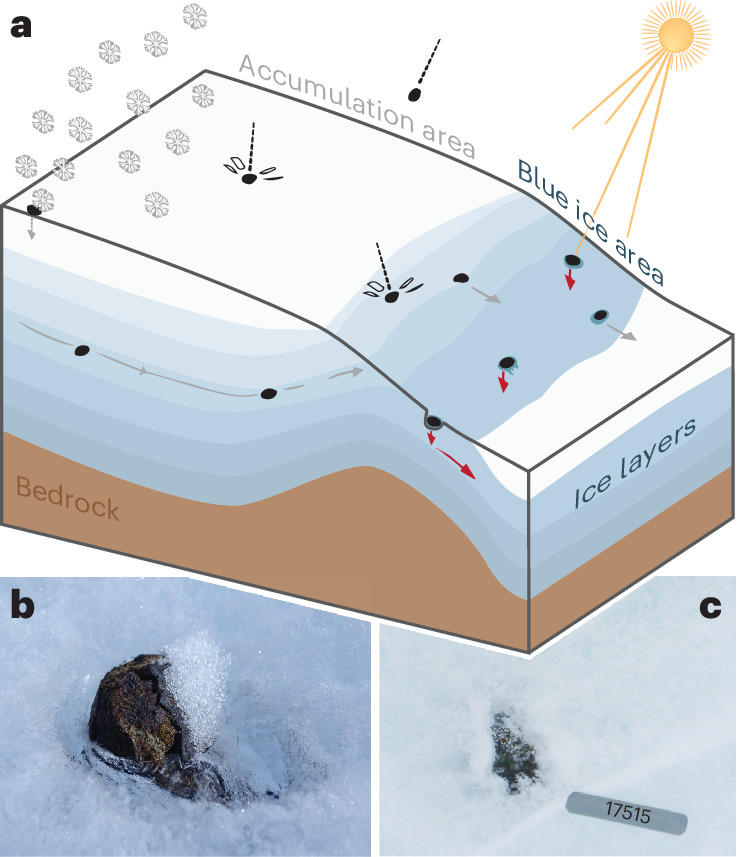
Antarctic meteorites in blue ice areas. **a**, Schematic representation of the meteorite concentration mechanism, with a supply of meteorites through ice flow and direct infall and loss of meteorites through melting from the surface into the ice (sinking, red arrows). The sinking of meteorites is caused by (increased) warming of the dark meteorites (especially those with high metal contents and thermal conductivity) under solar radiation, causing the underlying ice to melt, and hence the meteorite to sink into the ice. **b**, The Hutchison Icefield 18033 meteorite (49 g) collected as part of the Lost Meteorites of Antarctica Project^10,24^. **c**, Meteorite MIL 07710 (147 g) fully enclosed in ice, collected as part of the Antarctic Search for Meteorites (ANSMET) programme (the number in the photo is used for documentation in the field). A column of clear, bubble-free ice above the meteorite was observed during the field mission (transparent on photograph), indicating that the meteorite sunk through melting underlying ice that refroze as superimposed ice above the sample^16^. Credit: **b**, Katherine Joy, Lost Meteorites of Antarctica Project^10^^,^^24^; **c**, Ralph Harvey, ANSMET programme.

**Fig. 2:**
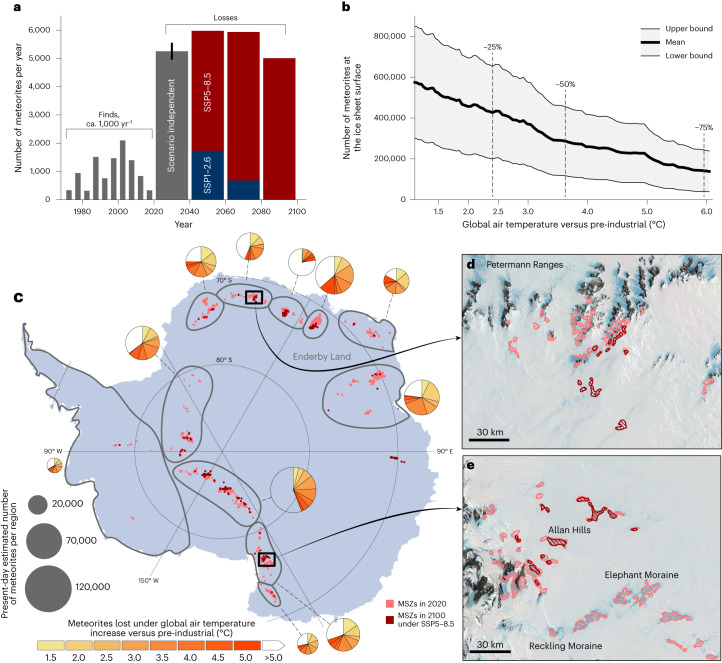
Projected evolution of meteorites in Antarctica under climate warming. **a**, Antarctic meteorite finds up to 2020 (that is, including January or February 2019, not including 2020) documented in the Meteoritical Bulletin^17^ (averaged over 5 yr intervals) and predicted future loss rates (averaged over 20 yr intervals) for a low-emissions scenario (Shared Socio-economic Pathway (SSP)1-2.6) and a high-emissions scenario (SSP5-8.5) (Supplementary Fig. 1). Estimates for the two emissions scenarios start to deviate from 2052; therefore loss rates for 2020–2040 are averaged for the two scenarios with the error bar representing the lower and upper estimates. **b**, The projected number of meteorites remaining at the ice sheet surface in relation to global air temperature increase with respect to pre-industrial levels (1850–1900; Supplementary Fig. 3). The graph displays the average estimate (bold line) and both the lower and upper bounds (grey shading; Methods), and indicates under which temperature increase 25%, 50% and 75% of the meteorites are lost. **c**, Continent-wide estimate of meteorite stranding zones (MSZs) in 2020 and in 2100 under SSP5-8.5 (both exaggerated with buffers of 10 km for visual clarity). The pie charts show the number of meteorites lost under global air temperature increases relative to pre-industrial values (colour scale) for the regions outlined in grey. In other parts of the Antarctic continent^31,32^ (that is, in regions that are not within grey boundaries), the total estimated numbers of meteorites are negligible (~0.5% of all meteorites in 2020). **d**, Unexplored meteorite stranding zones in the Petermann ranges in 2020 (pink) and 2100 (under SSP5-8.5, red). Potential new areas that appear are mostly snow covered (Methods). Background data are false-colour Landsat satellite images^33^. **e**, Identified meteorite stranding zones in the Allan Hills and Elephant Moraine area. The Allan Hills meteorite stranding zone (~1,800 meteorite finds so far) is projected to persist under a warming climate, while those at Elephant Moraine (~2,500 meteorite finds so far) and Reckling Moraine (~150 meteorite finds so far) are projected to disappear before 2100 under SSP5-8.5. Credit: **d**,**e**, Landsat Image Mosaic of Antarctica (LIMA) project.

Meteorites are unique samples of extraterrestrial bodies and provide crucial information on the origin and evolution of our Solar System^1,2^. Antarctica is the world’s most prolific site for collecting meteorites, with more than 60% of all ~80,000 meteorites ever found on Earth being collected at the surface of the ice sheet. Antarctic meteorites are found in blue ice areas, which are atypical zones (~1% of the Antarctic surface area) where layers of snow and ice are removed from the surface through a combination of ice flow processes and local meteorological conditions, exposing meteorites that were once embedded in the ice^1,3^. Not all blue ice areas contain meteorites: only where processes interact favourably, a concentration of meteorites is built up over tens to hundreds of thousands of years, resulting in so-called meteorite stranding zones (Fig. 1a)^4–6^. Meteorites found in Antarctica are a few centimetres in diameter on average, but are easily detectable given their visual contrast with the underlying ice^7,8^. Over past decades, an average of ~1,000 meteorites per year have been collected through numerous field campaigns (Fig. 2a) and the potential of Antarctic meteorites remains far from exhausted: a data-driven approach^9^ recently identified over 600 meteorite-rich areas in Antarctica. Many of the identified meteorite stranding zones are not yet (fully) explored, and an estimated 300,000 to 850,000 meteorites remain to be collected from the surface of the ice sheet (Fig. 2b)^9^.

Once exposed at the surface, meteorites can stay there for thousands of years due to stagnant ice flow and the lack of weathering in the cold, dry conditions^5,6^. While most of the indicators for the presence of meteorites—for example, ice flow velocity, elevation, mountains—are thought to be stable on multidecadal to centennial timescales, the concentration of meteorites is also directly influenced by temperature^4,9,10^. Even when temperatures are well below zero, meteorites, with their characteristic dark crust, warm when exposed to solar radiation^11^ and can melt the underlying ice. The warmed meteorite generates a small water melt pocket below the stone, resulting in a surface depression that deepens over time into a hole, which (in conjunction with refreezing meltwater) results in the disappearance (‘sinking’) of the meteorite from the surface (Fig. 1)^10,12,13^. The sensitivity of meteorite presence to temperature is apparent from various independent lines of evidence:

1. Field observations: entrapped meteorites have been found covered by superimposed (refrozen) ice after the meteorite sank into the ice (Fig. 1)^14–16^.
2. Data on meteorite retrieval locations^9,17^ indicate that almost no meteorites (<1% of all finds) are found in locations where surface temperatures of the ice are higher than −9 °C even very rarely (this near-maximum value of the surface temperature is the 99th percentile of 19 years of 8 day averages derived from satellite observations). Moreover, in situ observations at a much finer temporal resolution indicate that air and ice temperatures rarely exceed −5 °C for more than a few minutes at Antarctic meteorite stranding surfaces^18^.
3. Empirical and experimental studies of meteorite heating show that the sinking of meteorites occurs when the downward meteorite motion caused by melting into the underlying ice exceeds the local ablation rate^10,12^.
4. Thermodynamical modelling suggests that meteorites can sink into the ice with air temperatures above −10 °C (ref. ^4^).
5. Data-driven meteorite-site classifications^9^ indicate that near-maximum surface temperatures are an important predictor for the presence of meteorites.

Hence, the temperature susceptibility of meteorites at the surface of the ice could cause meteorite stranding zones to disappear under changing climatic conditions^4,9^.

To quantify the loss of Antarctic meteorites, we used a machine learning algorithm that predicts the presence of meteorites^9^ forced with dedicated regional climate model simulations (Methods). The machine learning algorithm captures interactions between different predictors of meteorite presence by estimating a multidimensional density distribution of observations of these different predictors (for example, ice flow velocity, surface temperature; Methods). Forcing the algorithm with future surface temperatures not only eliminates places that will become too warm for meteorites to be found in future climate conditions, but also considers interactions with other processes (for example, the ice flow velocity). While examining these interactions, the algorithm identifies locations for which future conditions become substantially different from current conditions at places where meteorites were recovered.

We found that in the coming decades, independent of the emissions scenario used (SSP1-2.6 or SSP5-8.5; Supplementary Fig. 1), ~5,000 meteorites yr^−1^ disappear from the surface of the Antarctic ice sheet in response to present warming conditions (Fig. 2a and Supplementary Fig. 1). This rate outpaces the rate at which Antarctic meteorites are found by about a factor of five (Fig. 2a). The estimated meteorite losses under the low- and high-emissions scenarios only start to deviate in the second half of the century (that is, after 2050). We estimated meteorite losses independent of these emissions scenarios by comparing the losses directly with the wide range of potential temperature increases captured under SSP5-8.5. The Antarctic continent-wide meteorite losses are strongly correlated with the increase in global air temperature: 5,100 to 12,200 meteorites (~1–2% of all current meteorites) are lost from the surface of the ice sheet for every tenth of a degree in temperature increase (*r* = −0.946 to −0.968 (Supplementary Fig. 2), the uncertainty range stems from the range in precision and sensitivity estimates of the machine learning algorithm; Methods). This fragile state can also be related to climate policy targets. If global warming is limited to 1.5 to 2.0 °C compared with global pre-industrial levels (Supplementary Fig. 3), the loss of meteorites can be constrained to between 9 and 20% compared with 2020. However, under current policies (that result in an estimated global warming of approximately 2.6 to 2.7 °C)^19,20^, 28–30% of the meteorites become unrecoverable. This share increases to 35% under scenarios with 3 °C of warming and 55% under 4 °C of warming. Under the high-emissions scenario (SSP5-8.5), 76% of the meteorites are lost by the end of this century and only 150 meteorite stranding zones (Methods) with an area of 3,180 km^2^ would remain, representing a decrease of 76% in the number of zones and of 78% in their areal extent (Fig. 2c–e).

The projected meteorite losses are not uniform across the continent. For some of the known dense meteorite collection areas^17^, we project that up to 50% of the total number of meteorites could be lost from the ice surface before 2050 (Supplementary Table 1). One example of these sensitive areas is the Grove Mountains in East Antarctica, a prime meteorite collection site where already more than 12,000 meteorites have been recovered^21^. In the promising, yet largely unexplored, Enderby Land region in East Antarctica (Fig. 2c), similar losses are projected, with 50% of meteorites disappearing before 2054. Data show that at present, the largest concentrations of meteorites are found at elevations between 1,800 and 2,000 m (refs. ^17,22^), where an 88% reduction is forecasted in the number of retrievable meteorites by the end of the century under the high-emissions scenario (Supplementary Fig. 4). Only at elevations above 2,500 m will the meteorite losses be lower than 50% (Supplementary Fig. 4). Hence, to preserve the unique information contained in Antarctic meteorites, ongoing meteorite losses not only call for fast action, but also a (global) coordination to secure the most vulnerable samples in areas that are particularly exposed to meteorite loss (for example, low-elevation meteorite stranding zones such as the Hutchison Icefield). At present, decisions on which areas to visit are largely made according to the availability of logistical support and national government science priorities^7^. In the field, meteorites are often found by human visual identification during grid searches, conducted either on foot or by snow mobile^8^. To increase retrieval rates of such labour-intensive operations, we suggest a major international effort to revisit known sites or access unexplored sites with larger searching teams over the next 10–15 years. Leveraging recent developments in robotics (for example, unoccupied aerial vehicle observations^23^ in harsh environments), as well as high-resolution modelling, could increase the efficiency and the extent of recovery operations, although the development of robust, scalable methods are very challenging in the extreme Antarctic^4,7,24^. Moreover, these techniques might allow the detection of some samples under ice or transient snow cover. Snow cover can be expected to be more prevalent in a warming climate^25–27^ and results in even more meteorites becoming unrecoverable, but this process was not considered here when estimating meteorite losses (Supplementary Section 2).

The ongoing loss of Antarctic meteorites is a consequence of climate change. Despite the delayed response of the interior of the Antarctic ice sheet to climate change in terms of ice melt (with temperatures remaining well below zero, even with several degrees of warming), meteorites are affected even by very minor (decimal) increases in surface temperatures during exceptionally warm events, which are expected to occur more frequently in the future^28^. Rapidly and purposefully collecting all meteorites is necessary to preserve the information on our Solar System that each additional sample contains: for example, information on the emergence of life on Earth through the delivery of water and organic matter, and how the Moon was formed^2,29^. A concerted effort would be similar in spirit to what is currently done in ice core research, where ice samples collected from vanishing, yet unique, glaciers—such as the few remaining tropical glaciers—are stored in long-term archives^30^. Ultimately, however, the only way to preserve the remaining unrecovered Antarctic meteorites is to rapidly reduce greenhouse gas emissions.

## Methods

Potential meteorite locations were identified using a machine learning algorithm (for details, see ref. ^9^) that relies on observations of ice flow velocity^34^, surface temperature^35^, radar backscatter^36^ and surface slope^37^. The temperature observations used to develop the classifier consist of the 99th percentile of the 19 yr (2001–2020) distribution of 8 day averaged surface temperature observations (that is, near-maximum temperature) of the Moderate Resolution Imaging Spectroradiometer (MODIS)^35^. To project the future temperature evolution, we used the climate model Modèle Atmosphérique Regional (MAR)^38^ at 35 km resolution (see Supplementary Section 3). In these dedicated high-resolution simulations, we fixed the extent of blue ice areas over time (by fixing the albedo). The output of MAR consists of daily surface temperature estimates, which we averaged to obtain 8 day estimates. From these data, for each year from 2020 to 2100, we retrieved the 99th percentile of the distribution of surface temperatures of the preceding 19 years. We then computed temperature anomalies with respect to the reference period 2001–2020 and added these anomalies to the observed temperatures (Supplementary Fig. 5).

We estimated the number of meteorites by converting the number of 450 m pixels that were identified as potential meteorite sites by the machine learning algorithm. We used an estimated precision of the classifier of 0.47–0.81 and an estimated sensitivity of 0.74–0.48 for the lower and upper bounds, respectively^9^. To derive absolute numbers, we used the fact that there are five meteorite finds per positive 450 m pixel (directly derived from the 12,906 meteorites that have been found over the 2,554 450 m pixels used to develop the classifier)^9^. For the lower bound, we did not consider any newly appearing meteorite stranding zones with respect to the reference year 2020. The physical understanding of the meteorite concentration mechanism indicates that there is temporally asymmetric behaviour regarding the (dis)appearance of meteorites (accumulating meteorites takes thousands of years, while they can be lost in a matter of years)^5,8,18^. However, for the upper estimate of the number of meteorites on the continent, we did not discard the limited number of newly appearing meteorites in existing blue ice areas and their near vicinity^9,39^. By doing so, we tend to overestimate the number of meteorites remaining on the ice sheet. A visual inspection of the newly appearing meteorite stranding zones showed that the algorithm identifies locations that are mostly snow covered (for example, Fig. 2d). Other uncertainties that result in meteorite losses higher than those predicted here are related to climate model uncertainties and the assumption that temperatures at meteorite locations did not change between the moment of collection (Fig. 2a) and the observational period (2001–2019). These processes are discussed in more detail in Supplementary Section 2. For both the upper and lower bounds, we subtracted the number of meteorites that have already been collected from the total number of meteorites throughout the century by: (1) excluding the locations that intersected with location data of meteorite finds; and (2) subtracting 32,307 meteorites from the estimates to account for the meteorite finds without (reliable) location information. Unless indicated as range, all presented values refer to the average between the upper and lower bounds. Loss rates (Fig. 2a) were estimated by fitting a piecewise linear function to the average number of meteorites over time. The fitting was performed using linear least squares (Supplementary Fig. 1).

## Online content

Any methods, additional references, Nature Portfolio reporting summaries, source data, extended data, supplementary information, acknowledgements, peer review information; details of author contributions and competing interests; and statements of data and code availability are available at 10.1038/s41558-024-01954-y.

### Supplementary information

Supplementary Information Supplementary Figs. 1–7, Table 1, text and references.

## Data Availability

All data needed to evaluate the conclusions in the paper are present in the paper and/or the Supplementary Information. Additional data related to this paper are available via Zenodo at 10.5281/zenodo.10579625 (ref. ^40^). Data used in this study comprise: (1) the MEaSUREs InSAR-Based Antarctica Ice Velocity Map, Version 2, available through NASA National Snow and Ice Data Center Distributed Active Archive Center (NSIDC DAAC)^34^; (2) MODIS/Terra Land Surface Temperature data, available through NASA EOSDIS Land Processes DAAC^35^; (3) RAMP AMM-1 SAR Image Mosaic of Antarctica, Version 2, available through NASA NSIDC DAAC^36^; (4) the Reference Elevation Model of Antarctica, available from the Polar Geospatial Center^37^; (5) blue ice area outlines^39^ and (6) geoid heights^41^, both part of the data package Quantarctica available through the Norwegian Polar Institute^42^; (7) MEaSUREs Antarctic Boundaries, Version 2, available through NASA NSIDC DAAC^31,32^; (8) the Landsat Image Mosaic of Antarctica, available through the United States Geological Survey^33^; (9) meteorite finding locations and (10) outlines of dense collection areas, available through the Meteoritical Society’s Meteoritical Bulletin Database^17^; and (11) the TanDEM-X PolarDEM of Antarctica, available through the repositories of the German Aerospace Center^22^.

## Code availability

Code constructed for the data analyses, Fig. 2 and Supplementary Figs. 1–7 is available via Zenodo at 10.5281/zenodo.10589098 (ref. ^43^).
