# Supplementary material for: Antarctic meteorites threatened by climate warming
Source: Nat Clim Chang. 2024 Apr 8;14(4):340–3. doi: 10.1038/s41558-024-01954-y (PMC11006603; doi:10.1038/s41558-024-01954-y)
Supplement: Supplementary file 1 — Supplementary Figs. 1–7, Table 1, text and references. [file 41558_2024_1954_MOESM1_ESM.pdf]

nature climate change

Brief Communication https://doi.org/10.1038/s41558-024-01954-y

Antarctic meteorites threatened by climate warming

In the format provided by the
authors and unedited

Supplementary materials – Table of contents

1. **Supplementary Figures and Tables**
    - **Figure S1:** Projected number of meteorites at the ice sheet surface until 2100 under two emissions scenarios
    - **Figure S2:** Correlation between the global air temperature increase and the Antarctic meteorite loss
    - **Figure S3:** Global near-surface air temperature changes over time with respect to pre-industrial values
    - **Figure S4:** Histogram of surface elevation at predicted meteorite finding locations
    - **Figure S5:** Near-maximum temperature changes over time with respect to 2020
    - **Table S1:** Projected meteorite losses in dense collection areas

2. **Uncertainties**
    - **Figure S6:** Near-maximum temperatures compared to average temperatures

3. **Climate model projections**
    - **Figure S7:** Comparison between modelled and observed temperatures

References for supplementary material

1. Supplementary Figures and Tables

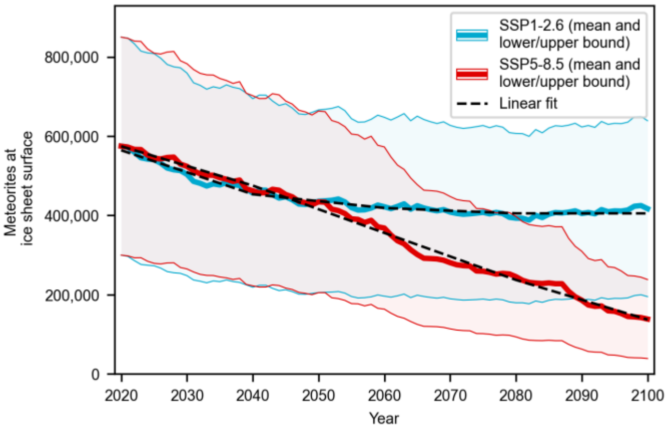

Figure S1: Projected number of meteorites at the ice sheet surface until 2100 under two emissions scenarios. The piecewise linear function is fitted using linear least squares, forcing negative slopes throughout the century. The estimated slopes of the piecewise linear function represent the loss rates for periods of 20 years (Figure 2A). Differences between SSP1-2.6 and SSP5-8.5 before mid-century are related to the internal variability of the climate model (see "3. Climate model projections" in Supplementary Materials). The projections under the different emissions scenarios start to deviate in 2052, when the difference of estimated meteorites between the low-emissions and high-emissions scenario becomes larger than 2% of the estimated meteorites in the low-emissions scenario.

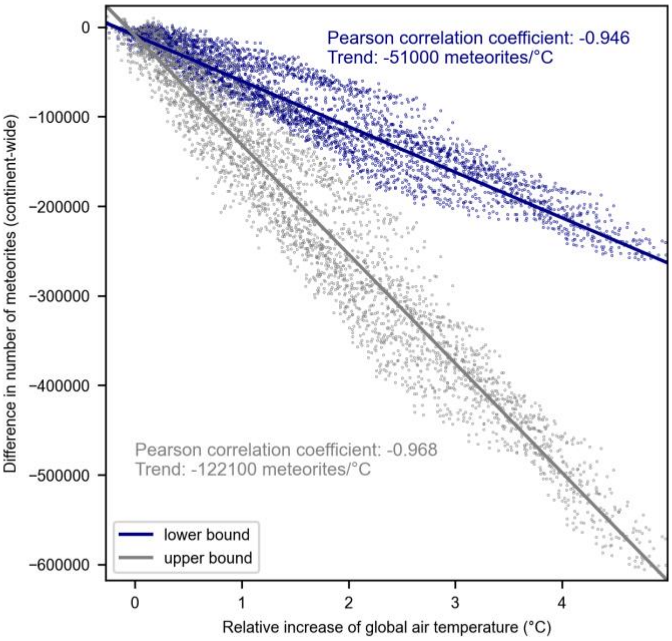

Figure S2: Correlation between the global air temperature increase and the Antarctic meteorite loss for the lower-bound scenario and the upper-bound (see Figure 2, Figure S1 and Methods). These values are computed by comparing the temperatures and the number of meteorites at any possible interval between 2020 and 2100, resulting in 3240 datapoints. The trends are computed by a linear least squares estimation.

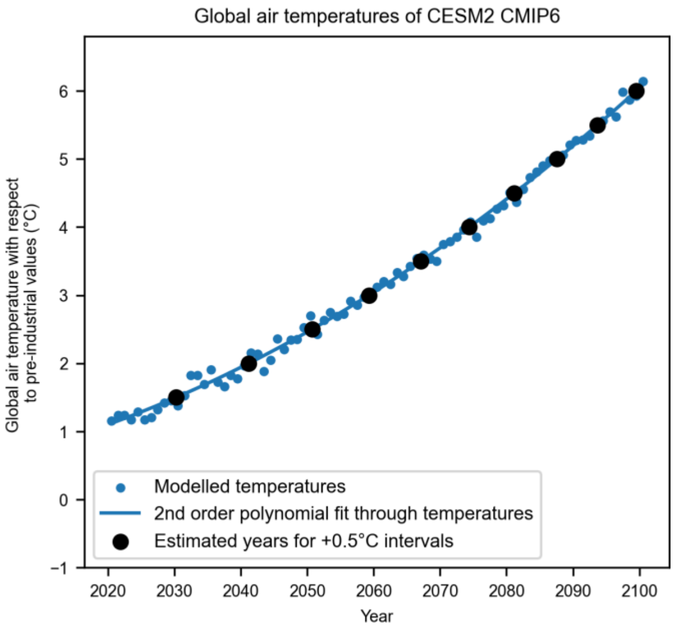

Figure S3: Global near-surface air temperature changes over time with respect to pre-industrial values. The data consists of near-surface air temperatures, averaged over the entire globe, from the Community Earth System Model 2 (CESM2) using the high-emissions scenario (SSP5-8.5)[1]. The comparison to pre-industrial levels (defined as 1850-1900 in the literature[2]) has been made in the same manner as for the near-maximum temperature changes over time (Figure S5). We fitted a 2nd order polynomial through the data from 2020 until the end of the century and shifted the polynomial (and the here shown modelled temperatures) so that in 2020 the global air temperatures are +1.1 °C with respect to pre-industrial values (https://berkeleyearth.org/data/). From the 2nd order polynomial fit through the modeled temperature changes, we estimated the years that correspond to a +1.5°C, +2.0°C, +2.5°C, and other warming levels relative to pre-industrial levels. From this smooth fit, we also estimated the temperature increases for each year, from which we constructed the x-axis of Figure 2B that displays the global air temperature increase.

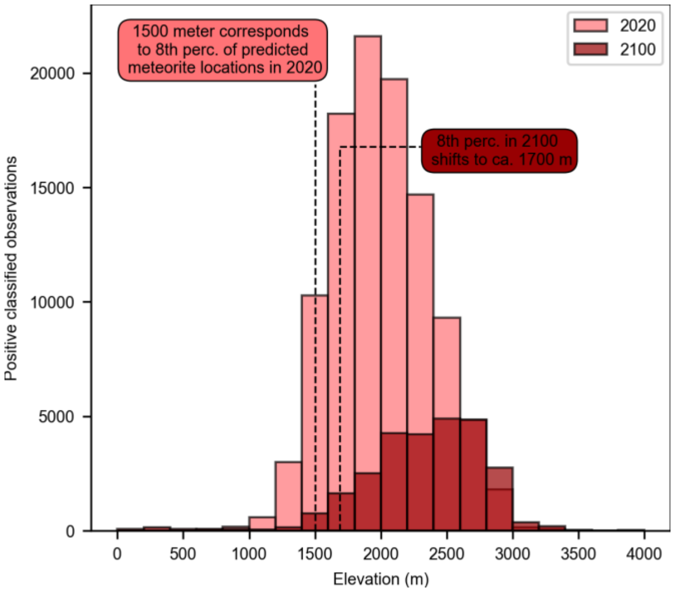

Figure S4: Histogram of surface elevation at predicted meteorite finding locations (excluding known locations where meteorites have been collected). The surface elevation is

extracted from the Digital Elevation Model (DEM) observed by the TanDEM-X (TerraSAR-X add-on for Digital Elevation Measurement) satellite at a resolution of 90 meter[3], where the ellipsoidal heights of the DEM are transformed to orthometric heights using approximated geoid heights provided in the data package Quantarctica[4,5]. We obtained the values at the gridded positive classified observation locations through bilinear interpolation. The values of 2100 consist of the average per bin of the two scenarios: (i) where new meteorites can appear with respect to 2020 and (ii) where no new meteorites can appear (see Methods). The rising temperatures will lead to a shift of the lower elevation limit at which meteorites can be found. The reported threshold of 1500 meters, which has been previously used to select potential meteorite stranding zones[6], corresponds to the present-day 8th percentile of predicted meteorite locations. This 8th percentile will be about 200 meters higher by the end of the century under a high-emissions scenario.

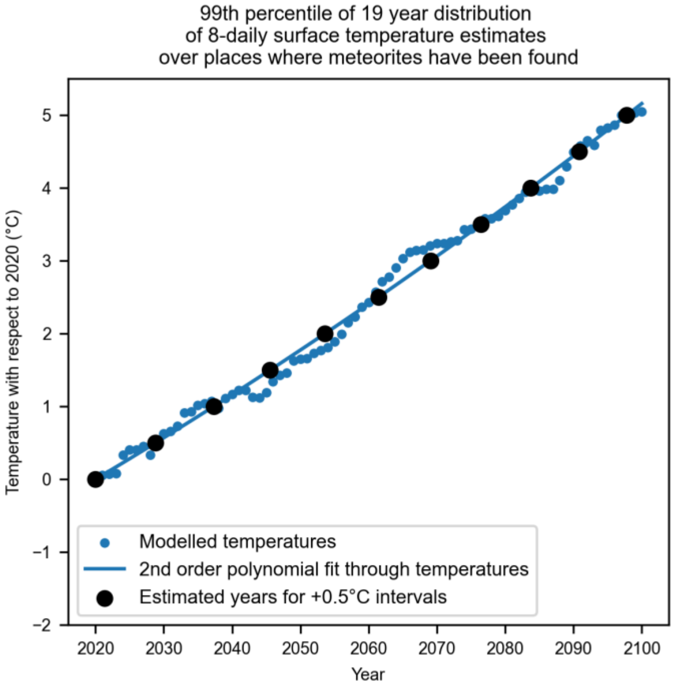

Figure S5: Near-maximum temperature changes over time with respect to 2020. The years correspond to the last year of a 19-year period (i.e., 2020 corresponds to the 99th percentile of 8-daily surface temperatures in the period 2001-2020).

Table S1: Projected meteorite losses in dense collection areas listed by the Meteoritical Society[7]. Table indicates the official name of the area, the predicted number of meteorites per square kilometre (representative for 2020) in the area as delineated in the data provided by the Meteoritical Society ("-" corresponds to no predicted meteorites within the outlined area), and the year in which 50% of the total number of meteorites (including those already collected) are projected to be lost. NB, the number of meteorites per square kilometre is not a direct output of the classifier, which just predicts a probability on the presence meteorites[8]. We use the quantity here to evaluate the accordance between the outlined dense collection areas and our predictions. Hence, the estimated meteorite densities reflect both errors in the predictions and in the outlined dense collection area and should not be used to prioritize certain regions.

Name of Dense Collection Area	Area (km^2)	Predicted number of meteorites per km^2 (see caption)	Estimated year in which 50% of meteorites are lost (under SSP1-2.6)	Estimated year in which 50% of meteorites are lost (under SSP5-8.5)
Lomonosov	16.7	1.0	>2100	2025
Finger Ridge	314.6	2.7	2030	2029
Mount Baldr	7.6	1.5	2030	2032
Odell Glacier	264.7	9.2	2031	2033
Amundsen Glacier	240.2	1.3	2042	2037
Hutchison Icefield	467.4	1.5	2031	2040
Grove Mountains	963.7	5.9	2064	2048
Mount Walton	16.3	20.3	2066	2049
Beckett Nunatak	63.2	10.6	2033	2049
Outpost Nunatak	33.9	9.8	2066	2055
Lonewolf Nunataks	464	0.6	2061	2056
Bates Nunataks	386.3	2.1	2053	2057
Bowden Névé	96.5	6.9	2066	2057
Nodtvedt Nunataks	203.2	1.2	2079	2058
Roberts Butte	7.7	15.2	2074	2058
Cumulus Hills	249.4	6.6	>2100	2059
Meteorite Hills	638.1	4.3	2056	2060
Mount DeWitt	44.7	12.8	2061	2061
Miller Butte	7	20.8	2074	2062
Outer Recovery Icefields	448.4	4.6	>2100	2062
Reckling Peak	492.3	6.5	2067	2062
Buckley Island	213.0	2.8	2100	2063
Taylor Glacier	28.9	6.4	>2100	2064
Mount Cranfield	57.3	9.1	2096	2064
Patuxent Range	4651.6	1.0	>2100	2064
Stewart Hills	15.2	12.2	>2100	2066
Pecora Escarpment	2936.2	5.9	>2100	2069
David Glacier	860.9	2.7	>2100	2070
Steingarden Nunataks	457.3	7.1	>2100	2074
Geologists Range	1025.0	2.2	2099	2076
Elephant Moraine	1598.8	5.1	>2100	2076
Szabo Bluff	84.4	16.2	>2100	2076
LaPaz Icefield	1663.8	8.9	>2100	2077
Frontier Mountain	104.3	17.2	>2100	2081
Dominion Range	1030.5	4.5	>2100	2081
Queen Alexandra Range	439.3	7.1	>2100	2089
Wisconsin Range	1591.8	2.4	>2100	2090
Miller Range	1202.4	9.9	>2100	2091
Lewis Cliff	223.6	8.4	>2100	2092
Thiel Mountains	2564.4	3.3	>2100	2097
Mount Fleming	12.3	11.8	>2100	2100
D'Angelo Bluff	31.1	3.5	>2100	>2100
Allan Hills	3276.4	3.8	>2100	>2100
MacKay Glacier	380.2	3.7	>2100	>2100
Graves Nunataks	1605.4	2.7	>2100	>2100
Mount Prestrud	98.6	6.2	>2100	>2100
Mount Wisting	142.0	3.5	>2100	>2100
Roberts Massif	480.4	2.9	>2100	>2100
Mount Howe	96	5.0	>2100	>2100
Klein Glacier	20.1	0.3	>2100	>2100
Grosvenor Mountains	2385.1	3.5	>2100	>2100
Larkman Nunatak	154.9	10.4	>2100	>2100
MacAlpine Hills	124.9	8.2	>2100	>2100
Mount Pratt	96.2	4.9	>2100	>2100
Sandford Cliffs	83.6	8.5	>2100	>2100
Gardner Ridge	22.7	2.8	>2100	>2100
Scott Glacier	92.6	17.3	>2100	>2100
Devils Glacier	23.4	-		-
Mount Crean	3.6	-		-
Inland Forts	9.7	-		-
Purgatory Peak	2.4	-		-
Patriot Hills	0.5	-		-
Derrick Peak	59.0	-		-
Tentacle Ridge	262.6	-		-

2. Uncertainties

We identify three possible limitations to the estimated numbers (e.g., Figure 2) that could potentially increase the meteorite loss significantly (i.e., we consider our loss estimates to be conservative). Firstly, the climate model output does not directly indicate an increase in heat-wave-like events as would be expected in a warmer climate and has been demonstrated with climate model projections[9]. The projected near-maximum surface temperatures (here defined as the 99th percentile of the 19-year distribution of 8-daily mean surface temperatures) over areas where meteorites have been found shifts only with 0.74 degrees for every 1 degree that the yearly average surface temperature over the Antarctic continent increases (r=0.993; Figure S6). This relationship could potentially be explained by a cap of surface temperatures in MAR at 0°C (by definition, if the temperature is above 0°C, the excess energy is used for melt, and the temperature returns to 0°C). So, over time, there must be more places where the surface temperature can no longer rise beyond 0°C.

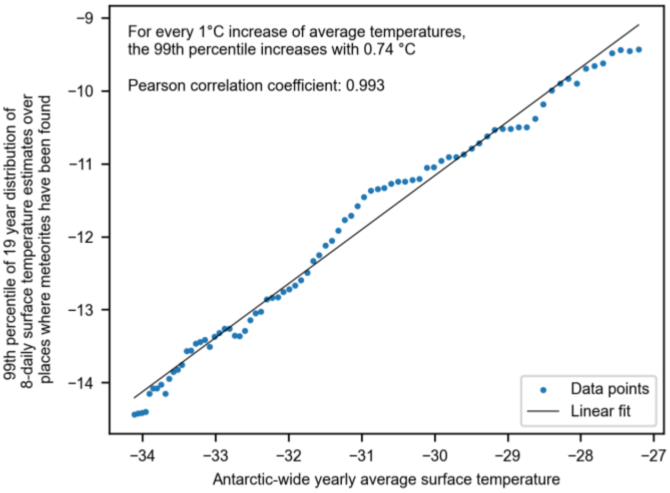

Figure S6: Near-maximum temperatures compared to average temperatures. Correlation between the near-maximum surface temperatures at all known meteorite finding locations[7] (y-axis) and the Antarctic-wide yearly average surface temperatures (x-axis).

Next, we do not consider potential changes of the exposure of blue ice. In the high-elevation blue ice areas where meteorites are typically found, the variability in the extent of blue ice is determined by temporal (and spatial) variability in the surface mass balance[10]. Increases in surface mass balance reduce the exposed area of ice in time spans of weeks, while decreases in surface mass balance will result in sublimation of the firn layer until blue ice is exposed, a process that takes much longer[11] and results in limited expansion of the blue ice, as the observed horizontal mass-balance in blue ice areas is steep[10]. Hence, if the blue ice extent at high elevation (i.e., where sublimation is the dominant ablative process) changes, it is more likely that blue ice becomes snow-covered, causing meteorites exposed at the surface to become invisible[10,12]. Moreover, it is unlikely that young (new) blue ice areas contain a high concentration of meteorites as the build-up of such a concentration takes tens to hundreds of thousands of years[13].

Another limitation that is not captured in the estimated range is related to the fact that the meteorite classification algorithm does not consider a time component – we assume here that its predictions are valid for the end of a 19-year interval of surface temperature observations. However, many of the meteorite finds used to train the algorithm are older than this 19-year period, where we know that surface temperatures were lower. This results in a classifier that is

lenient regarding surface temperatures, i.e., with a hypothetical classifier that considers the time-component, the upper bound of the estimated loss range would be even higher. Moreover, with rising temperatures in the past decades, the build-up of meteorite concentrations could also be affected, because meteorites can sink before emerging to the surface. As meteorites get close to the surface (20-50 cm), the rock is affected by sunlight that penetrates through the ice[14]. This effect is enhanced in meteorites that are metal-rich (i.e., iron meteorites) and might explain the relatively small share of iron meteorites in the Antarctic collection, leading to the hypothesis of a hidden layer of iron meteorites under the surface of meteorite stranding zones[14,15].

3. Climate model projections

We use the Modèle Atmosphérique Régional (MAR)[16] to project surface temperature changes. MAR is a polar-oriented regional climate model frequently used over the Greenland and Antarctic ice sheets. The model is forced every six hours at its boundaries by the surface pressure, air temperature and humidity, wind (u,v) components, sea surface temperature and sea ice of the Community Earth System Model 2 (CESM2) using a low-emissions and a high-emissions scenario (SSP1-2.6 and SSP5-8.5, respectively). CESM2 has special parametrisations to better represent polar climates[17]. Although CESM2 has a high equilibrium climate sensitivity, it enables us to explore a large range of climate warming.

Since none of the climate models, including MAR, correctly simulate the location of all the blues ice areas, we forced their location in the model by prescribing an albedo representative of blue ice areas. This albedo is based on a blue ice index (percentage of blue ice for each 35-km pixel of MAR) and represents a linear transition between no blue ice (albedo of 0.92, representing fresh snow) and 100% blue ice (albedo of 0.55, representing blue ice in MAR). The blue ice extent is based on rasterizing blue ice outlines (provided in the data package Quantarctica[5,18]) to a 200-meter-resolution grid. We fixed the albedo values to not change over time, even in the event of fresh snowfall or melting. Prescribing the albedo makes it possible to calculate the energy balance and the resulting surface temperature representative of blue ice. However, the fixed albedo prevents the incorporation of albedo-feedback processes in MAR, which would enhance the surface warming significantly[19], implying that the estimated surface temperatures are probably a lower bound of potential increases.

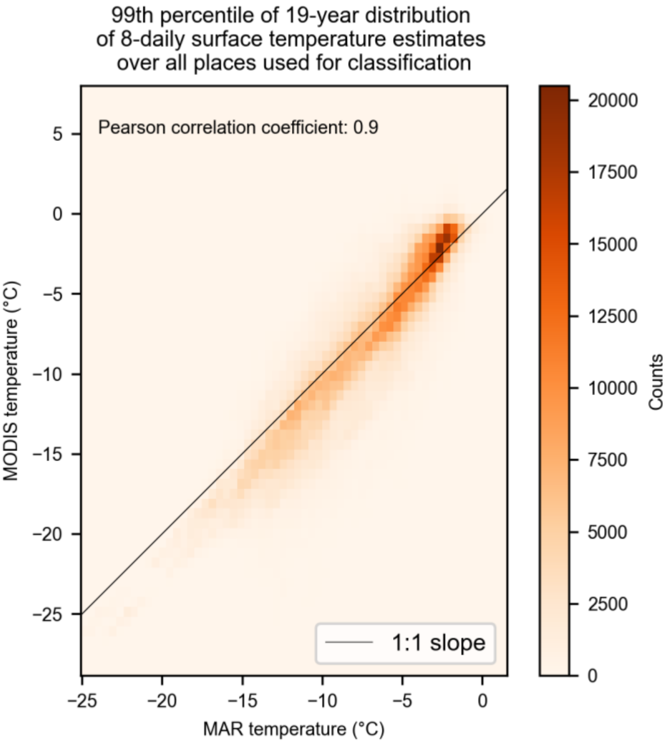

Figure S7: Comparison between modelled and observed temperatures over all places used for the classification of meteorite stranding zones (i.e., places where meteorites have been found, and potential meteorite-finding places consisting of blue ice areas and their near vicinity, represented by a 1-km buffer around blue ice outlines[18]).

We extract the temperature anomalies (see Methods) for each 450-meter pixel used in the

meteorite-stranding-zone classifier by bilinear interpolation. Despite the fairly coarse resolution of MAR, the modelled surface temperatures compare well to the observed temperatures that were used to construct the meteorite-zones classifier, i.e., the 99th percentile of the 19-year (2001-2020) distribution of 8-daily surface temperatures. The good correlation between the modelled and observed temperatures (r=0.90), and the near-one-to-one slope in Figure S7 indicates that the anomalies that we add to the observations are not biased toward either end of the temperature range.

References for supplementary material

1. Danabasoglu, G. *et al.* The Community Earth System Model Version 2 (CESM2). *Journal of Advances in Modeling Earth Systems* **12**, e2019MS001916 (2020).
2. IPCC. *Climate Change 2023: Synthesis Report. A Report of the Intergovernmental Panel on Climate Change. Contribution of Working Groups I, II and III to the Sixth Assessment Report of the Intergovernmental Panel on Climate Change [Core Writing Team, H. Lee and J. Romero (Eds.)]*. (Geneva (Switzerland), 2023).
3. Wessel, B. & Huber, M. TanDEM-X - PolarDEM - Antarctica, 90m. [Dataset] German Aerospace Center (DLR) https://doi.org/10.15489/9JHR18JEPI65 (2020).
4. Foerste, C. *et al.* EIGEN-6C4 The latest combined global gravity field model including GOCE data up to degree and order 2190 of GFZ Potsdam and GRGS Toulouse. [Dataset] GFZ Data Services https://doi.org/10.5880/ICGEM.2015.1 (2014).
5. Matsuoka, K. *et al.* Quantarctica, an integrated mapping environment for Antarctica, the Southern Ocean, and sub-Antarctic islands. *Environmental Modelling & Software* **140**, 105015 (2021).
6. Harvey, R. The Origin and Significance of Antarctic Meteorites. *Geochemistry* **63**, 93–147 (2003).
7. Meteoritical Bulletin Database. Available at https://www.lpi.usra.edu/meteor (Meteoritical Society, accessed on November 15, 2022). (2022).
8. Tollenaar, V. *et al.* Unexplored Antarctic meteorite collection sites revealed through machine learning. *Science Advances* **8**, eabj8138 (2022).
9. Feron, S. *et al.* Warming events projected to become more frequent and last longer across Antarctica. *Scientific Reports* **11**, 19564 (2021).
10. Bintanja, R. & Van Den Broeke, M. R. The climate sensitivity of Antarctic blue-ice areas. *Annals of Glaciology* **21**, 157–161 (1995).

11. Ligtenberg, S. R. M., Lenaerts, J. T. M., Van Den Broeke, M. R. & Scambos, T. A. On the formation of blue ice on Byrd Glacier, Antarctica. *Journal of Glaciology* **60**, 41–50 (2014).
12. Brown, I. C. & Scambos, T. A. Satellite monitoring of blue-ice extent near Byrd Glacier, Antarctica. *Annals of Glaciology* **39**, 223–230 (2004).
13. Haack, H., Schutt, J., Meibom, A. & Harvey, R. Results from the Greenland Search for Meteorites expedition. *Meteoritics & Planetary Science* **42**, 1727–1733 (2007).
14. Smedley, A. R. D., Evatt, G. W., Mallinson, A. & Harvey, E. Solar radiative transfer in Antarctic blue ice: spectral considerations, subsurface enhancement, inclusions, and meteorites. *The Cryosphere* **14**, 789–809 (2020).
15. Evatt, G. W. *et al.* A potential hidden layer of meteorites below the ice surface of Antarctica. *Nature Communications* **7**, 10679 (2016).
16. Kittel, C. *et al.* Diverging future surface mass balance between the Antarctic ice shelves and grounded ice sheet. *The Cryosphere* **15**, 1215–1236 (2021).
17. Dunmire, D., Lenaerts, J. T. M., Datta, R. T. & Gorte, T. Antarctic surface climate and surface mass balance in the Community Earth System Model version 2 during the satellite era and into the future (1979–2100). *The Cryosphere* **16**, 4163–4184 (2022).
18. Hui, F. *et al.* Mapping blue-ice areas in Antarctica using ETM+ and MODIS data. *Annals of Glaciology* **55**, 129–137 (2014).
19. Jakobs, C. L., Reijmer, C. H., van den Broeke, M. R., van de Berg, W. J. & van Wessem, J. M. Spatial Variability of the Snowmelt-Albedo Feedback in Antarctica. *Journal of Geophysical Research: Earth Surface* **126**, e2020JF005696 (2021).
